# An imConvNet-based deep learning model for Chinese medical named entity recognition

**DOI:** 10.1186/s12911-022-02049-4

**Published:** 2022-11-21

**Authors:** Yuchen Zheng, Zhenggong Han, Yimin Cai, Xubo Duan, Jiangling Sun, Wei Yang, Haisong Huang

**Affiliations:** 1 Medical College, Guizhou University, Guiyang, 550025 Guizhou China; 2 Key Laboratory of Advanced Manufacturing Technology, Ministry of Education, Guizhou University, Guiyang, 550025 Guizhou China; 3 Guiyang Hospital of Stomatology, Guiyang, 550002 Guizhou China

**Keywords:** Named entity recognition, Convolutional neural network, Chinese electronic medical records, BiLSTM-CRF, BERT

## Abstract

**Background:**

With the development of current medical technology, information management becomes perfect in the medical field. Medical big data analysis is based on a large amount of medical and health data stored in the electronic medical system, such as electronic medical records and medical reports. How to fully exploit the resources of information included in these medical data has always been the subject of research by many scholars. The basis for text mining is named entity recognition (NER), which has its particularities in the medical field, where issues such as inadequate text resources and a large number of professional domain terms continue to face significant challenges in medical NER.

**Methods:**

We improved the convolutional neural network model (imConvNet) to obtain additional text features. Concurrently, we continue to use the classical Bert pre-training model and BiLSTM model for named entity recognition. We use imConvNet model to extract additional word vector features and improve named entity recognition accuracy. The proposed model, named BERT-imConvNet-BiLSTM-CRF, is composed of four layers: BERT embedding layer—getting word embedding vector; imConvNet layer—capturing the context feature of each character; BiLSTM (Bidirectional Long Short-Term Memory) layer—capturing the long-distance dependencies; CRF (Conditional Random Field) layer—labeling characters based on their features and transfer rules.

**Results:**

The average F1 score on the public medical data set yidu-s4k reached 91.38% when combined with the classical model; when real electronic medical record text in impacted wisdom teeth is used as the experimental object, the model's F1 score is 93.89%. They all show better results than classical models.

**Conclusions:**

The suggested novel model (imConvNet) significantly improves the recognition accuracy of Chinese medical named entities and applies to various medical corpora.

## Background

Name entity recognition (NER) is a term that refers to the task of extracting proper nouns or other named entities from text [1]. NER is frequently used in machine translation, emotion analysis, information retrieval, and other fields as a critical step in converting unstructured data to structured data during information extraction. It is a topic of discussion in natural language processing (NLP) [2]. With the development of current medical and computer technology [3–7], medical information management becomes perfect. Modern medical management systems play an essential role in successfully preserving and managing text-based data such as electronic medical records (EMRs) and medical reports [8]. The constant accumulation of data enables the analysis of large-scale medical text data. How to fully exploit the resources of information of these medical data has always been the subject of research by many scholars. Using NLP to mine medical texts, particularly EMRs, has become a hot spot of cross-research in medicine and artificial intelligence.

Certain methods of medical NER are based on traditional machine learning. The commonly used models include Hidden Markov Models [9], Maximum Entropy [10], Support Vector Machine (SVM) [11, 12], and Conditional Random Field (CRF) [13–15]. Tang et al. [16] combined the advantages of CRF and SVM and suggested the Structural Support Vector Machine (SSVM) algorithm to explore its use in clinical NER tasks. Ye Feng, Chen Yingying [17], and colleagues extracted three common entities from Chinese EMRs using the CRF method: disease, clinical symptoms, and surgical operation. The three entities' best F1 values were 92.67%, 93.76%, and 95.06%, respectively. However, feature engineering requires a significant effort when using traditional machine learning [18]. Most work is spent on data preprocessing to produce a high-quality NER effect [19], making NER inefficient and costly. Simultaneously, medical data problems from high-dimensional sparse data and limited scalability render medical NER based on traditional machine learning worthless [20].

Deep learning has rapidly been developed and entered the public's vision to address these problems. It has achieved considerable success in various fields, including image and speech processing. Simultaneously, it has been increasingly applied to NLP tasks for deep learning-based medical NER. Lample [21] et al. proposed an approved model named BiLSTM-CRF model, and then it widely used in medical NER tasks [22, 23]. NER of Chinese EMR has its particularity. Chinese EMRs have a complicated structure, a large number of different types of entities, and a certain domain of particularity. Medical proper nouns have unique naming rules [24], but traditional NER methods map words to one-hot encoding, which cannot express word polysemy. As a result, scholars propose to solve this problem using the pre-training model to represent words.

Among the earliest used pre-training models are word vector training with the word2vec method [25, 26], ELMo algorithm [27], and GloVe algorithm [28]. In 2018, Google published a paper suggesting BERT [29], which immediately showed significant success in 11 NLP tasks and ushered in scholars' widespread usage of BERT model. Many scholars have combined BERT and classic models BiLSTM-CRF to greatly improve the accuracy of NER [30, 31]. Due to the success of BERT, pre-training models are beginning to incorporate a significant number of parameters to improve performance. However, increasing the number of model parameters introduces many problems, including higher and higher requirements for computing power, a longer time for model training, and, in some situations, the worse performance of models with many parameters. Albert [32, 33] suggested improving these problems by reducing the overall number of parameters, accelerating the training speed, and increasing the model effect.

The abovementioned models and methods have gradually become the mainstream of NER in the medical field. Numerous models based on the Transformer [34] neural network emerge in an infinite number of ways. The Transformer has replaced RNN and CNN as the significant backbone architecture in the field of NLP. Simultaneously, it has made a significant contribution to the field of computer vision. In recent years, most publications in CV field have been based on Transformer, and the convolutional neural network has begun to fade from center stage. Will Transformer take the place of the convolutional neural network? The proposal of ConvNeXt model answers. Facebook AI research and UC Berkeley published an article in January 2022: a ConvNet for the 2020s [35], suggesting a pure convolutional neural network. Several experimental comparisons revealed that ConvNeXt has faster reasoning speed and higher accuracy than Swing Transformer under the same FLOPS. CNN has also been utilized for NER, compared with NLP field [36–38]. Even though the traditional CNN has obvious computational advantages, the traditional CNN's terminal neurons can only extract a fraction of the information contained in the input text following convolution. Further convolution layers must be added to gather context information, resulting in ever-deeper networks, an increasing number of parameters, and easy overfitting. Strubel et al. [39] proposed applying dilated convolution (IDCNN) to NER to address this issue. IDCNN outperformed BiLSTM in terms of effect and training speed on CoNLL-2003 dataset.

As a result of the preceding discussion, it is worthwhile to investigate if CNN remains useful for improving NER accuracy. This paper trains the CNN model to improve NER effect, beginning with the strategies and ideas in the Transformer, investigating ways to improve the accuracy of traditional convolutional neural networks using mainstream classical models, and creating the model imConvNet to improve the accuracy of Chinese medical NER.

## Method

### Theoretical principle

#### BERT model

BERT is a pre-training model and is not limited by a single-directional language model, unlike earlier pre-training models. It pre-trains the model using a “masked language model” (MLM) and then builds it using a deep bidirectional Transformer component. Finally, it provides a deep bidirectional language representation that is capable of fusing left and right context information, hence expanding the model's representation capability. Additionally, with only one additional output layer, the pre-trained BERT representation may be fine-tuned to generate state-of-the-art models for a variety of tasks.

**Fig. 1:**
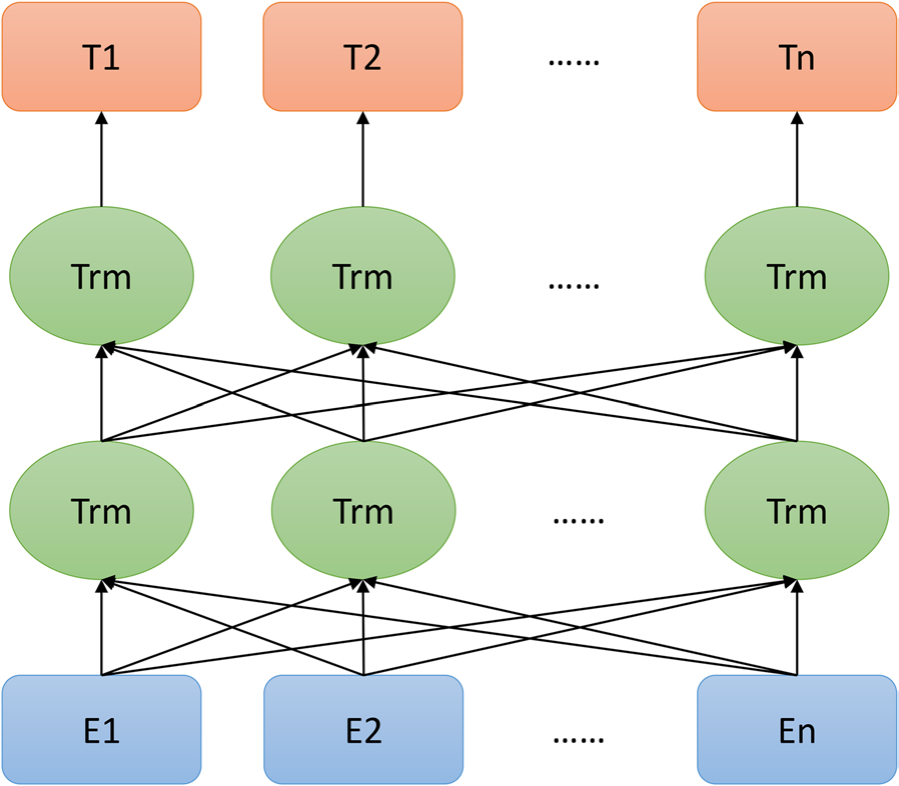
Basic structure diagram of BERT

BERT's core structure is a transformer. The main structure of BERT is constructed by stacking multi-layer Transformer structures, as illustrated in Fig. 1.

#### Long short-term memory

**Fig. 2:**
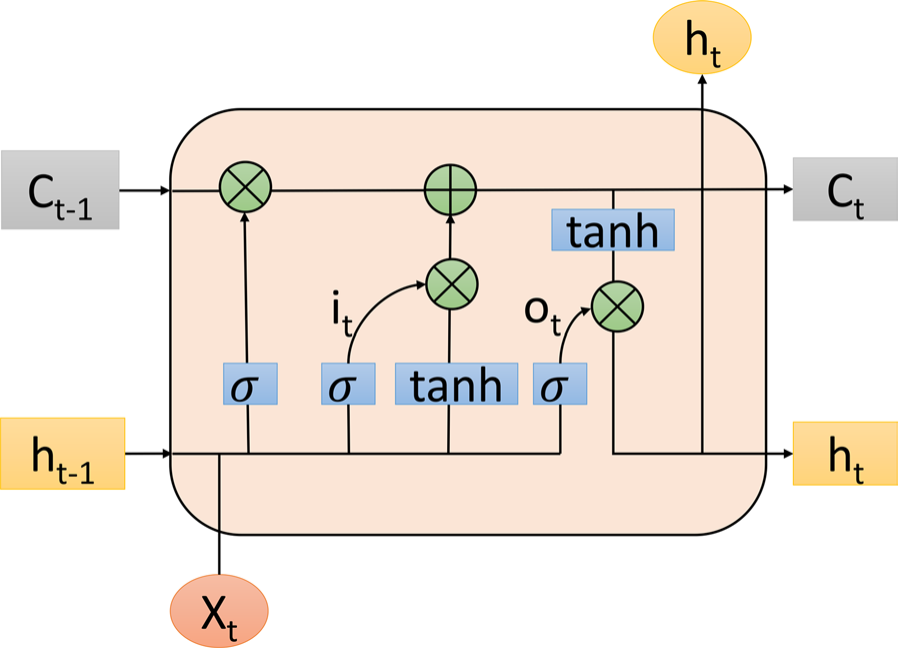
Basic structure diagram of LSTM

The Long Short-Term Memory (LSTM) network [40] is a more advanced model of the recurrent neural network (RNN). It is capable of capturing long-distance dependencies and remembering long-term information for the purpose of modeling context information in NLP tasks. Through training, LSTM can learn which information to retain and discard. Figure 2 illustrates its structure:

Where ${X}_{t}$ represents the word input at time t, ${C}_{t}$ represents the cell state, $\widetilde{{C}_{t}}$ represents the temporary cell state, ${h}_{t}$ represents the hidden layer state, ${i}_{t}$ represents the memory gate and ${o}_{t}$ represents the output gate. Relevant parameters are updated to the following formula:

1 $$f_{t} = \sigma \left( {W_{f} \cdot \left[ {h_{t - 1} ,x_{t} } \right] + b_{f} } \right)$$

2 $$i_{t} = \sigma \left( {W_{i} \cdot \left[ {h_{t - 1} ,x_{t} } \right] + b_{i} } \right)$$

3 $$\widetilde{{C_{t} }} = \tanh \left( {W_{C} \cdot \left[ {h_{t - 1} ,x_{t} } \right] + b_{C} } \right)$$

4 $$C_{t} = f_{t} *C_{t - 1} + i_{t} *\widetilde{{C_{t} }}$$

5 $$o_{t} = \sigma \left( {W_{o} \cdot \left[ {h_{t - 1} ,x_{t} } \right] + b_{o} } \right)$$

6 $$h_{t} = o_{t} *\tanh \left( {C_{t} } \right)$$

However, it is incapable of encoding information from back to front. This issue can be resolved by combining forward and backward LSTMs into Bi-LSTM to accurately represent bidirectional semantic dependencies.

#### Conditional random field

Conditional random field (CRF) is a sequence labeling model that considers the relationship between output labels, effectively models the sequence relationship between final prediction labels, and improves the accuracy and reasonableness of prediction results.

Given a set of input sequences $X=({X}_{1},{X}_{2},{X}_{3},\dots ,{X}_{n})$, the conditional probability distribution model of the output sequence $y=({y}_{1},{y}_{2},{y}_{3},\dots ,{y}_{n})$ can be acquired, and the score of each label can be obtained so that the label with the highest score can be selected as the final output label. The score is calculated as follows:

7 $$S\left( {X,y} \right) = \mathop \sum \limits_{i = 0}^{n} A_{{y_{i} ,y_{i + 1} }} + \mathop \sum \limits_{i = 0}^{n} P_{{i,y_{i} }}$$

Among them, ${A}_{i,j}$ is the possibility of transition from label ${y}_{i}$ to label ${y}_{j}$. ${y}_{0}$ and ${y}_{n}$ are the first and last labels of a sentence. They are added to a list of possible labels. Then, using the softmax function, the normalized probability of label sequence y is determined, and the label sequence y is the output:

8 $$p\left( {y{|}X} \right) = \frac{{e^{{s\left( {X,y} \right)}} }}{{\mathop \sum \nolimits_{{\overline{y} \in {\Upsilon }_{X} }} e^{{s\left( {X,\overline{y}} \right)}} }}$$

Finally, the loss function (9) defined below is used to optimize the log-likelihood of the correct label sequence for CRF training, and Viterbi algorithm (10) is used to predict the optimal score as follows:

9 $$\log \left( {p\left( {y{|}X} \right)} \right) = s\left( {X,y} \right) - {\text{log}}\left( {\mathop \sum \limits_{{\overline{y} \in {\Upsilon }_{X} }} e^{{s\left( {X,\overline{y}} \right)}} } \right)$$

10 $$y^{*} = argmax_{{\overline{y} \in {\Upsilon }_{X} }} s\left( {X,\overline{y}} \right)$$

### Model building

#### imConvNet block

This section introduces the imConvNet layer's fundamental modules. Convolutional neural network (CNN) can effectively extract local characteristics from input data. It starts with a convolution layer as the network's basic element, which utilizes a convolution kernel with a small scale relative to the original data as the parameter and convolutes the input data using the convolution kernel according to the following formula:

11 $$y\left( n \right) = \mathop \sum \limits_{i = - \infty }^{\infty } x\left( i \right)h\left( {n - i} \right) = x\left( n \right)*h\left( n \right)$$

where $x(n)$ represents the input data and $h(n)$ represents the convolution kernel.

First, a depth-wise revolution is adopted to split the channels and regions in the convolution and perform layer by layer convolution calculation, as inspired by ConvNeXt model [35]. Assuming that the feature matrix of input word vector is ${D}_{f}\times {D}_{f}\times M$, the convolution kernel size is ${D}_{k}\times {D}_{k}\times M$, the output feature matrix is ${D}_{f}\times {D}_{f}\times N$, and the amount of parameters for standard convolutional layers is ${D}_{k}\times {D}_{k}\times M\times N$. The depthwise convolution is responsible for filtering, with a size of ${D}_{k}\times {D}_{k}\times 1$, M in total, and acts on each input channel. Therefore, the depthwise convolution parameter is ${D}_{k}\times {D}_{k}\times 1\times M$, which is 1/N of the standard convolution parameter, and a good balance between FLOPS and accuracy.

Then, using two convolutions with a size of $1\times 1$, GELU [41] is chosen as the activation function, and the idea of regularization is added to the activation. The calculation formula is as follows:

12 $$GELU\left( x \right) = xP\left( {X \le x} \right) = x\phi \left( x \right) = x*\frac{1}{2}\left[ {1 + {\text{erf}}\left( {\frac{x}{\sqrt 2 }} \right)} \right]$$

where erf is the error function:

13 $${\text{erf}}\left( x \right) = \frac{2}{\sqrt \pi }\mathop \smallint \limits_{0}^{x} e^{{ - t^{2} }} dt$$

Normalization can be achieved by substituting layer normalization (LN) [42] for the commonly utilized batch normalization (BN) in CNN, which is more appropriate for the field of NLP, accelerates network convergence, and reduces overfitting. It normalizes the different channels of the same sample. The calculation formula is:

14 $$y = \frac{x - E\left[ x \right]}{{\sqrt {Var\left[ x \right] + \varepsilon } }}$$

where: $E\left[ x \right] = \frac{1}{n}\mathop \sum \limits_{i = 1}^{n} x_{i}$, $Var\left[ x \right] = \frac{1}{n}\mathop \sum \limits_{i = 1}^{n} \left( {x_{i} - E\left[ x \right]} \right)^{2}$, $\varepsilon$ is used to prevent division by zero.

**Fig. 3:**
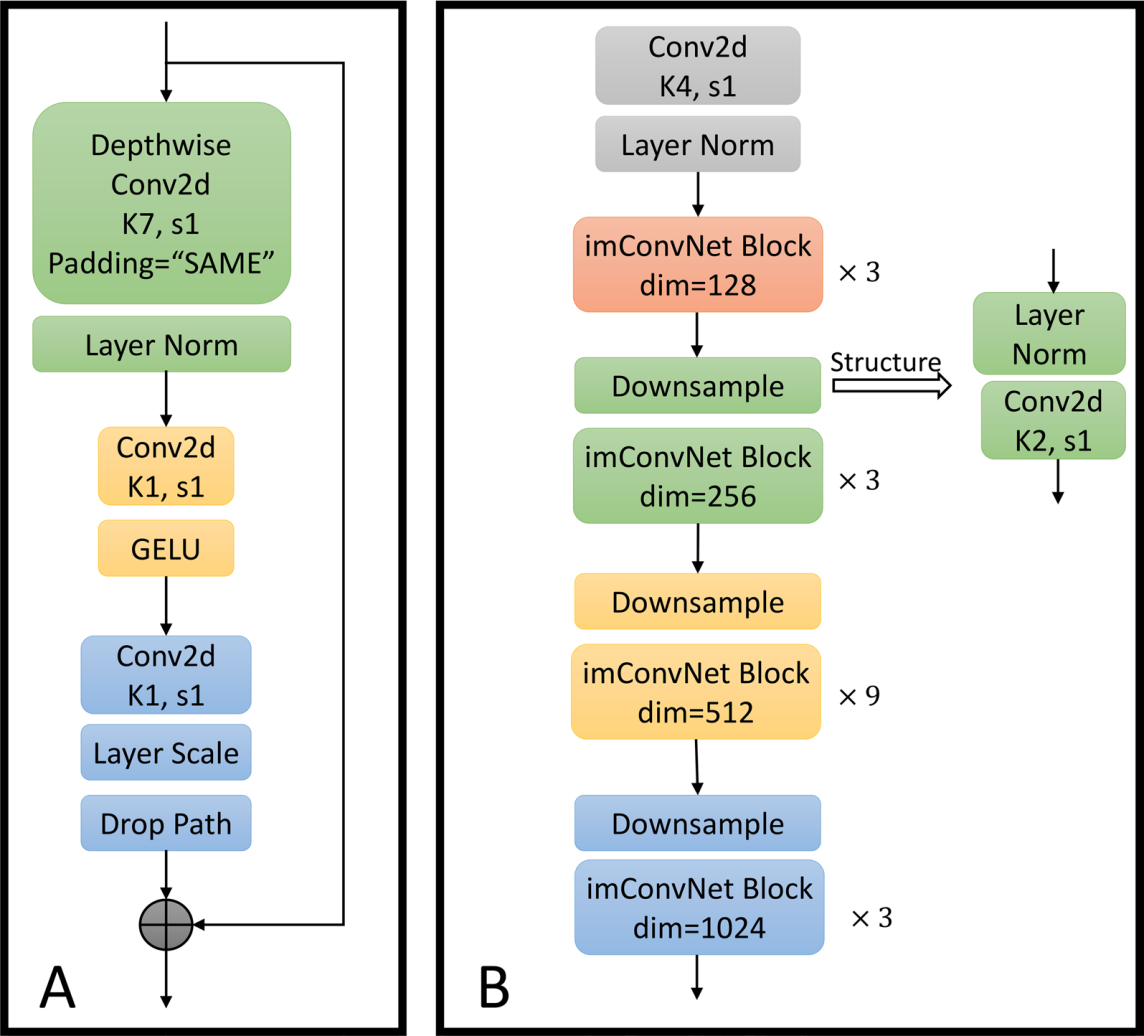
Basic structure diagram of imConvNet. (**A**) The structure of imConvNet Block; (**B**) the structure of imConvNet layer

The overall structure of the module is depicted in Fig. 3A.

#### imConvNet layer

This section describes the process of constructing the convolution layer. Many comparative experiments develop the ConvNeXt model's relevant parameters [35]. In this paper, we develop the whole module stack by utilizing the same stack ratio 3:3:9:3 of the tiny version. Rather than that, we modify the number of channels in each model's original input feature layer from 96, 192, 384, and 768 to 128, 256, 512, and 1024, which are more widely used in NLP. Then, in imConvNet layer, the module that initially implements local feature extraction utilizes a convolutional layer with a convolution kernel width of 4.

Additionally, we change the sliding step size 4 of this layer's convolution to 1 to make it acceptable for text feature extraction and then normalize it using LN (Layer Normalization) method. Simultaneously, downsampling is performed between each module, using a separate downsampling layer composed of a layer normalization and a convolution layer with a convolution kernel size of 2 and stride of 1. Finally, unlike the ConvNeXt, we record the result of each module's convolution, and the results of each module's convolution are spliced to obtain the output of the final imConvNet layer. The overall structure is depicted in Fig. 3B.

#### BERT-imConvNet-BiLSTM-CRF

**Fig. 4:**
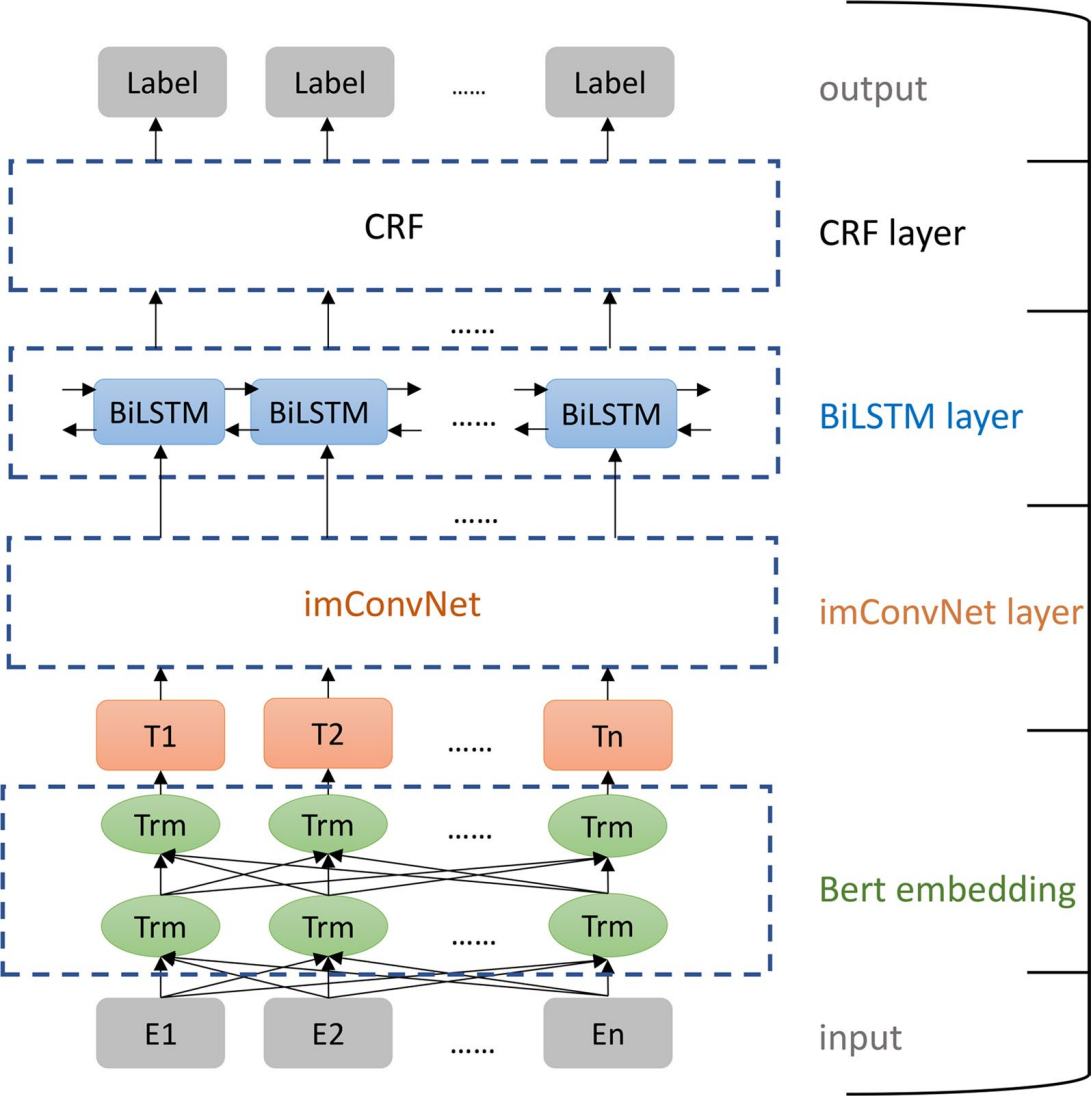
The overall structure of the model

In terms of the model's overall structure, we begin by using BERT pre-training model to obtain the word embedding vector according to the input text. To make the vector meet the imConvNet layer's usage conditions, we enhance the vector by one dimension before entering the imConvNet layer. Second, the processed word vector is passed via an imConvNet layer, which is utilized to extract the current word's local characteristics to acquire as much context information as feasible. Then, using BiLSTM layer, extract context-dependent long-range features and produce label predictions for all characters. Finally, the CRF layer constrains the output of the label to produce the final legal label. The overall structure of the model is described in Fig. 4.

### Evaluation metrics

The evaluation metrics, namely, Precision (P), Recall (R) and F1-measure (F1) are used to evaluate the performance of NER methods.

P is utilized to demonstrate the proportion of samples divided into positive examples that are actually positive examples. The calculation formula is described as follows:

15 $$P = \frac{TP}{{TP + FP}}$$

It reflects the prediction accuracy of the model for positive examples.

R denotes the number of positive examples divided into positive examples. The calculation formula is described as follows:

16 $$R = \frac{TP}{{TP + FN}}$$

It indicates the actual ability of the model to discriminate positive examples.

However, recall and precision are an indicator of trade-offs, when one of the values goes up, the other goes down and we need an indicator to balance them. F1-score represents a metric combining recall and precision using harmonic mean. The calculation formula is described as follows:

17 $$\frac{2}{F1} = \frac{1}{P} + \frac{1}{R}$$

When the F1-score is higher, the performance of the model is also better.

Where TP (True Positive) shows the number of positive classes predicted to be positive, FP (False Positive) describes the number of negative classes predicted to be positive, and FN (False Negative) demonstrates the number of positive classes predicted to be negative.

### Dataset

#### Yidu-s4k dataset

This dataset is derived from one of the evaluation tasks of the National Conference on Knowledge Graph and Semantic Computing CCKS 2019 [43] (China Conference on knowledge graph and Semantic Computing), which is the dataset of "NER for Chinese EMRs", Yidu Cloud Medicine manually edits that in accordance with the actual distribution of medical records. There are a total of 1000 real clinical medical record corpora, with six entities: (1) Disease and Diagnosis, medically defined diseases, and doctors' judgments on etiology, pathophysiology, classification and staging in clinical work, such as “直肠癌(rectal cancer)”; (2) Anatomical site, human anatomy where disease, symptoms and signs happen, such as “肝(liver)”; (3) Laboratory check, laboratory tests performed in clinical work, such as “CEA(carcinoembryonic antigen)”; (4) Image examination, such as “CT”; (5) Drug, specific chemicals used in disease treatment, such as “奥沙利铂(Oxaliplatin)”; (6) Operation, treatment such as excision, suture, performed by the doctor on the part of the patient's body, such as “胃癌切除术(gastrectomy for gastric cancer)”.

We used BIO labeling with 13 label types, as shown in Table 1.

**Table 1:** Yidu-s4k dataset entity format

Entity types	Labels	
Disease and diagnosis	B-disease	I-disease
Anatomical site	B-position	I-position
Laboratory check	B-LabCheck	I-LabCheck
Image examination	B-check	I-check
Drug	B-drug	I-drug
Operation	B-method	I-method

**Fig. 5:**
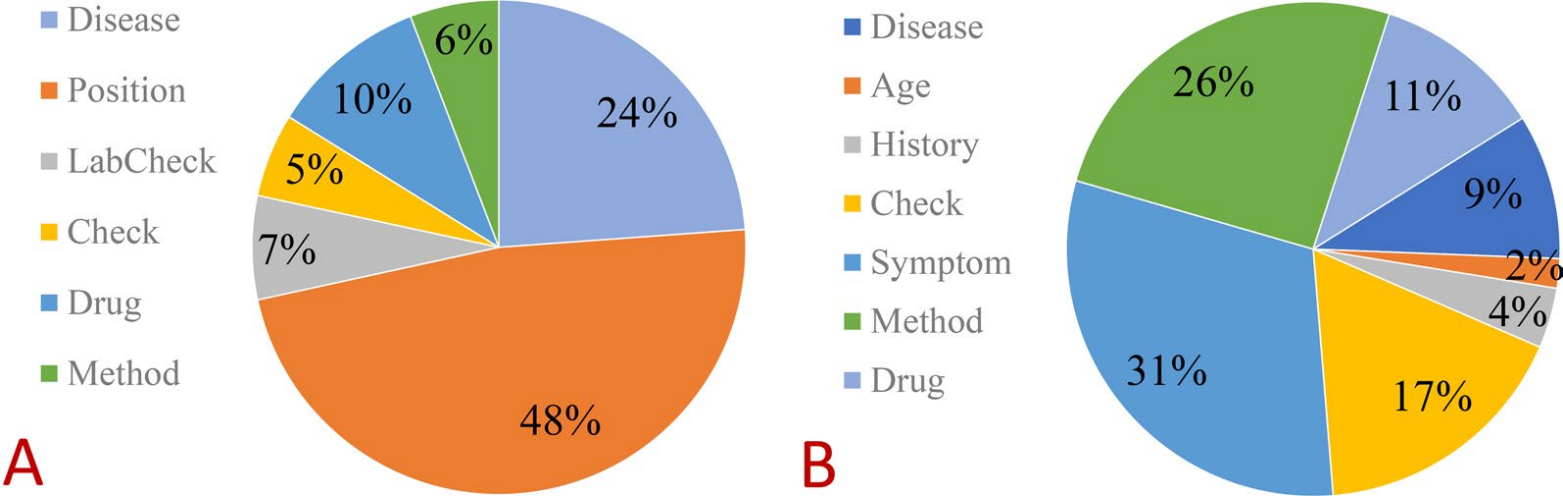
The number of **A** Yidu-s4k dataset entities and **B** clinical datasets of impacted wisdom teeth entities

The number distribution of each entity is displayed in Table 2 and Fig. 5A, reaching 17,637 entities.

**Table 2:** The number of Yidu-s4k dataset entities

Entity name	Numbers
Disease	4207
Position	8419
LabCheck	1195
Check	966
Drug	1822
Method	1028

#### Clinical dataset of impacted wisdom teeth

Impacted wisdom teeth, a prevalent and high-incidence disease in the field of stomatology, are critical for clinical research. However, the medical NLP datasets have not been used in this field. This clinical dataset on impacted wisdom teeth is a corpus of clinical medical records that have been sorted according to the actual medical records of impacted wisdom tooth extraction and patient examination reports from Guiyang Hospital of Stomatology. According to the experience and opinions of clinicians, seven types of entities were extracted: (1) Disease, types of impacted wisdom teeth, like “垂直阻生(vertical impacted wisdom teeth)”; (2) Symptom, clinical manifestations of impacted wisdom teeth, like “部分萌出(partially eruption)”; (3) Age, patient's age, like “20岁(20 years old)”; (4) History, patient's past history, like “糖尿病(diabetes)”; (5) Check, the patient's preoperative routine report, like “白细胞数目(white blood cell count)”; (6) Method, the process of removing impacted wisdom teeth, like “缝合牙龈(suture the gingiva)”; (7) Drug, specific chemicals used in disease treatment, like “替硝唑片(Tinidazole Tablets)”.

The clinical dataset of impacted wisdom teeth was obtained using BIO annotation format for entity annotation, as described in Table 3. The number distribution of each entity is displayed in Table 4 and Fig. 5B, reaching 14,095 entities.

**Table 3:** Clinical dataset of impacted wisdom teeth entity format

Entity types	Labels	
Disease	B-disease	I-disease
Symptom	B-symptom	I-symptom
Age	B-age	I-age
History	B-history	I-history
Check	B-check	I-check
Method	B-method	I-method
Drug	B-drug	I-drug

**Table 4:** The number of clinical datasets of impacted wisdom teeth entities

Entity name	Numbers
Disease	1339
Age	272
History	556
Check	2427
Symptom	4336
Method	3599
Drug	1566

## Results and discussion

In this paper, all the experiments are conducted on a single NVIDIA GeForce GTX 1650Ti GPU with 4 GB memory and carried out on the PyCharm platform using the python language. The relevant environment configuration required for the experiment is shown in Table 5. The data were divided randomly into a train set, a validation set, and a test set in the ratio of 6:2:2. The training was conducted with the parameters demonstrated in Table 6. Under the condition that each model's training parameters were consistent, the models proposed in this paper first used the yidu-s4k dataset and clinical dataset of impacted wisdom teeth, respectively, for training and then tested using a data set.

**Table 5:** Hardware and software environment

Device	Configuration
Operating system	Windows 10
Processor	Intel(R) Core (TM) i7-10750H CPU @2.60 GHz 2.59 GHz
GPU	GeForce GTX 1650Ti GPU with 4 GB memory
Framework	TensorFlow
Compilers	PyCharm
Scripting language	Python 3.7

**Table 6:** Hyper-parameters

Parameters	values
lstm_dim	256
batch_size	16
epoch	60
dropout_keep	0.5
Learning rate	0.001
optimizer	adam

**Fig. 6:**
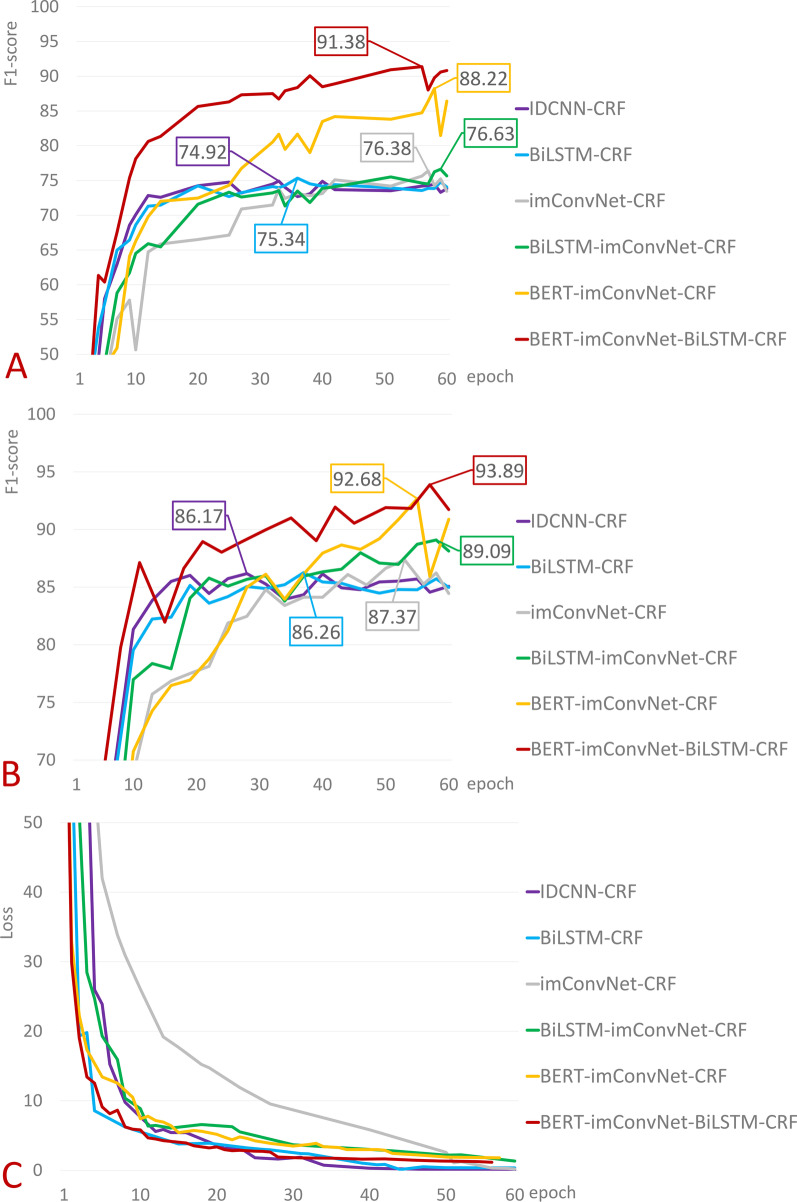
**A** The epoch and F1 relationship on the Yidu-s4k dataset. **B** The epoch and F1 relationship on clinical dataset of impacted wisdom teeth. **C** The epoch and Loss relationship on the Yidu-s4k dataset

On the yidu-s4k dataset, according to comprehensive F1 score results of different models shown in Fig. 6A and Table 7, the F1 score of classical BiLSTM-CRF model for NER is 75.34%, whereas F1 score of IDCNN-CRF model is 74.92%. Compared to original text proposed by IDCNN-CRF model, it does not reach F1 score mentioned in the text and surpasses BiLSTM model. The explanation for this could be that the author utilizes CoNLL-2003 dataset, which contains a large amount of data and a limited number of entity types and is unrelated to the medical field. We connect the constructed convolutional neural network imConvNet with CRF for NER, obtaining an F1 score of 76.38% for the model. This value is greater than BiSLTM-CRF model, and P and R increased by 0.86% and 1.24%, respectively. However, as shown in Fig. 6C, the figures of IDCNN-CRF and BiLSTM-CRF tend to stabilize between 20 and 30th epoch, while the figures of imConvNet-CRF does not tend to stabilize until around 50th epoch. It can be seen from the change of the loss function that imConvNet-CRF has a slower convergence speed compared to IDCNN-CRF and BiLSTM-CRF. Combining BiLSTM and imConvNet for NER, F1 score increased by 0.25–76.63%, and P and R increased by 1.93% and − 1.5%, respectively. BERT is a highly effective pre-training model derived from large-scale public corpus training. The pre-training Chinese BERT model is called directly to encode the new task's sentences. The F1 score of Bert-imConvNet-CRF model is significantly improved, reaching 88.22%. When compared with the previously discussed models, P, R, and F1 scores are increased by 12.5%, 10.7%, and 11.59%, respectively. When combining BiLSTM model to construct Bert-imConvNet-BiLSTM-CRF model, the maximum F1 score reached 91.38%, in which P, R, and F1 scores increased by 2.03%, 4.26%, and 3.16% respectively compared with the former. The results indicate that improved CNN model imConvNet obtained good results in the medical NER task. The three evaluation indexes of P, R, and comprehensive F1 scores improved slightly.

**Table 7:** Performance comparison of different models on the yidu-s4k dataset

Model	Evaluation index (%)	Entity type						Comprehensive value
		Disease	Position	LabCheck	Check	Drug	Method	
IDCNN-CRF	P(precision)	71.94	72.07	81.35	81.56	77.51	77.55	74.09
	R(recall)	70.88	78.83	76.67	76.25	72.38	78.35	75.77
	F1-score	71.41	75.30	78.94	78.81	74.86	77.95	74.92
BiLSTM-CRF	P(precision)	75.13	71.14	77.78	81.42	77.88	73.63	73.98
	R(recall)	72.16	80.06	76.36	78.93	72.93	76.29	76.75
	F1-score	73.62	75.34	77.06	80.16	75.32	74.94	75.34
imConvNet-CRF	P(precision)	72.45	74.79	77.06	72.26	81.06	76.77	74.84
	R(recall)	75.81	78.02	79.39	80.84	80.39	78.35	77.99
	F1-score	74.10	76.37	78.21	76.31	80.72	77.55	76.38
imConvNet-BiLSTM-CRF	P(precision)	74.53	76.19	77.78	81.60	78.84	81.77	76.77
	R(recall)	70.78	78.72	82.73	78.16	75.14	76.29	76.49
	F1-score	72.61	77.43	80.18	79.84	76.94	78.93	76.63
BERT-imConvNet-CRF	P(precision)	89.77	90.74	95.62	91.89	96.58	91.74	89.27
	R(recall)	89.38	86.95	94.70	97.14	97.35	90.97	87.19
	F1-score	89.57	88.81	95.16	94.44	96.96	91.35	88.22
BERT-imConvNet-BiLSTM CRF	P(precision)	92.36	92.44	97.32	95.45	98.42	95.48	91.30
	R(recall)	94.73	92.48	96.39	96.00	99.20	93.28	91.45
	F1-score	93.53	92.46	96.85	95.73	98.81	94.37	91.38

In terms of entity types, we horizontally compared the recognition impacts of various entities under different models and compared BERT-imConvNet-BiLSTM-CRF model finally constructed in this paper with IDCNN-CRF, BiLSTM-CRF, imConvNet-CRF, imConvNet-BiLSTM-CRF, and Bert-imConvNet-CRF. Compared to the models with the highest F1 score in the control group, the F1 score in the disease entity was increased by 3.96%, the position entity was increased by 3.96%, and the lab check entity was increased by 1.69%. It increased by 1.29% in the checking entity, 1.85% in the drug entity, and 3.02% in the method entity. It demonstrates that the new model's F1 score for each entity has been improved.

Table 8 shows the results of our method compared with previous representative systems on the yidu-s4k dataset [44–47]. The first system [44] used a domain-specific ELMo model, the encoder from Transformer (ET) as model’s encoder, and the CRF as the decoder, which achieved an F1 score of 85.59% on the yidu-s4k dataset. The second system [45] also used the ELMo model but the variant ELMo model uses Chinese characters as input, and then combine with the lattice LSTM model, achieved an F1 score of 85.02% on the yidu-s4k dataset. Jun et al. [46] combine the multi-level CNN with a simple attention mechanism, and also proposed a data augmentation method to expand the data volume, which achieved an F1 score of 85.13%. Ling et al. [47] achieved an F1 score of 85.16% by adopting the method of transfer learning and ensemble. Our approach achieved an F1 score of 91.38% which exceed the above models. It demonstrates that the effectiveness of our method is evident.

**Table 8:** Performance comparison between our approach and previous systems on the yidu-s4k dataset

Models	P (precision)	R (recall)	F1-score
ELMo-ET-CRF [44]	83.65	87.61	85.59
ELMo-lattice-LSTM-CRF [45]	84.69	85.35	85.02
ACNN [46]	83.07	87.29	85.13
FS-TL(Ensemble) [47]	–	–	85.16
Our approach	91.30	91.45	91.38

Simultaneously, as displayed in Table 9 and Fig. 6B, IDCNN-CRF model extracted entities from the clinical dataset of impacted wisdom teeth, and the comprehensive F1 score is 86.17%. BiLSTM-CRF model has an F1 score of 86.26%, which was increased by 0.09%. P and R were increased by 1.25% and − 1.21%, respectively. The imConvNet-CRF model has an F1 score of 87.37%, which was increased by 1.11%. P and R were increased by − 1.21% and 3.81%, respectively. The imConvNet-BiLSTM-CRF model has an F1 score of 89.09%, which was increased by 1.72%. P and R were increased by 3.79% and − 0.71%, respectively. After including BERT model, the model's influence was significantly improved. The comprehensive F1 score of BERT-imConvNet-CRF reached 92.68%, which was increased by 3.59%, while P and R were increased by 3% and 4.24%, respectively. Finally, by combining BERT, imConvNet, and BiLSTM, the comprehensive F1 score of the model reached 93.89% which was increased by 1.21%, and P and R were increased by 1.81% and 0.55%, respectively. While there are some fluctuations in the improvement of P and R, the F1 score is improving, and the performance change is consistent with that observed on the public dataset Yidu-S4K.

**Table 9:** Performance comparison of different models on a clinical dataset of impacted wisdom teeth

Models	P (precision)	R (recall)	F1-score
IDCNN-CRF	82.97	89.62	86.17
BiLSTM-CRF	84.22	88.41	86.26
imConvNet-CRF	83.01	92.22	87.37
imConvNet-BiLSTM-CRF	86.80	91.51	89.09
BERT-imConvNet-CRF	89.80	95.75	92.68
BERT-imConvNet-BiLSTM-CRF	91.61	96.30	93.89

In summary, the imConvNet model proposed in this paper has the value of improving the accuracy of entity extraction in the task of Chinese medical NER. We can extract text features with more context information and improve the capture of context long-distance dependent characteristics. The final BERT-imConvNet-BiLSTM-CRF model constructed applies to a variety of datasets in the field of Chinese medicine.

## Conclusion

The training and testing results on Yidu-S4K dataset demonstrate that pure convolutional neural network can still achieve good results in the task of Chinese medical NER. Although the classical BiLSTM model effectively extracts context characteristics, it has problems of lack of local spatial feature extraction. It can be replaced with the imConvNet convolutional neural network introduced in this paper, which extracts local features more efficiently than the traditional CNN model. Combining imConvNet and BiLSTM model yields better results than using only one model. To address the problem of a limited training corpus, we applied BERT pre-training Chinese model in this research. After incorporating BERT model, NER's F1 score significantly improved. Finally, we constructed Bert-imConvNet-BiLSTM-CRF model and trained it on our clinical corpus of impacted wisdom teeth, which also achieved better results. We will continue to adjust imConvNet model parameters in subsequent experiments to make it more suitable for Chinese medical NER and to enhance recognition accuracy and convergence speed and obtain good results. At the same time, we only conducted experiments on two medical datasets, and we will continue to test this model on more different datasets to improve the comprehensive performance and robustness of the model.

## Data Availability

The datasets (yidu-s4k) analysed during the current study are available in the Chinese EMR named entity recognition task in China Conference on Knowledge Graph and Semantic Computing in 2019 repository, https://www.biendata.xyz/competition/ccks_2019_1/. The data (Clinical dataset of impacted wisdom teeth) that support the findings of this study are available from Guiyang Hospital of Stomatology but restrictions apply to the availability of these data, which were used under license for the current study, and so are not publicly available. Data are however available from the authors upon reasonable request and with permission of Guiyang Hospital of Stomatology.

## Abbreviations

NER: Named entity recognition
NLP: Natural language processing
EMRs: Electronic medical records
LSTM: Long short-term memory
RNN: Recurrent neural network
CRF: Conditional random field
